# The Book World of Medicine and Science

**Published:** 1904-05-07

**Authors:** 


### The Book World of Medicine and Science.

Burdett's Hospitals and Charities, 1904, being the Year Book of Philanthropy and Hospital Annual. By Sir Henry Burdett, K.C.B. (London: The Scientific Press. Pp. 1,123. Price 5s.)

It is always with pleasure that we await the publication of this annual, for we have learned to look to it for enlightenment on many of the more urgent problems of the day which affect the philanthropical institutions of the empire. So far as the general arrangement is concerned, the present volume closely resembles its predecessors, and consists of a charity directory, prefaced by some 214 pages devoted to the discussion of various topics bearing on the management of hospitals and other charitable institutions. So far as the directory is concerned, there is not much to say beyond that it has been brought thoroughly up to date, and forms a complete guide, not only to the hospitals of this country, the colonies, and the United States, but also to the medical universities, colleges, and schools of the United Kingdom, dispensaries, asylums, convalescent homes, nursing institutions, sanatoria for consumption, missionary and religious societies, orphanages, homes for inebriates, and so forth.

The preliminary chapters contain, analysed and reduced to their simplest terms, some of the problems of the moment with which present-day charities are face to face. The first chapter is entitled "The Hospital Problem in the Metropolis," and deals with the great question of the hour—namely, the enormous growth of expenditure on reconstruction and rebuilding which has been of necessity entailed upon the hospitals in recent years. During the next decade the sum required for these purposes cannot be less, Sir Henry Burdett estimates, than £1,500,000, exclusive altogether of the large sums which have yet to be laid out in connection with the London Hospital. The redeeming feature is that when all this capital outlay is completed, the London hospitals should be able to continue their work with the maximum of efficiency for the next fifty years at least. Other questions dealt with in this portion of the volume concern hospital sites, the probable effects on existing medical schools of the development of the University of London, amalgamation of special hospitals, progress in administration of provincial hospitals, the cost of nursing departments and a quantity of other matters. A noteworthy chapter is that which deals with home and foreign missions. Attention is called to the fact that the cost of management of the various missionary societies varies from 34 per cent. to 2¼ per cent. of the total ordinary expenditure. It is further shown that the form in which the accounts of many of these societies is at present rendered offers insuperable obstacles to the formation of an idea as to the financial position and needs of a particular institution, and it is urged that every missionary society should therefore adopt an uniform system of book-keeping and analysis.

Apart from the preliminary portion of the volume, which provides a great deal of instruction pleasurably conveyed for anyone who takes a real interest in the administration of our charities, the directory portion is so full and technically complete that anyone desiring to ascertain the regulations or principles of management can find all that he wants from the pages of this volume. This point is not understood or the book would find its way on to the committee-room table of every hospital in the world.

Insanity in Everyday Practice. By E. G. Younger, M.D., M.R.C.P., &c. (London: Baillière, Tindall, and Cox. Pp. 190. Crown 8vo. Price 2s. 6d. net.)

For a good many years past the medical student has been compelled to attend lectures on mental disease, and to study insanity in the wards of a public asylum, by which means he is supposed to acquire some theoretical and practical acquaintance with one of the most difficult branches of his profession; but books on the subject suitable for the general practitioner have been few. Dr. Younger has done something to make that want less felt, and his book is exactly what he claims it to be: an outline chart rather than a detailed reference map. The vast majority of our general practitioners have neither the time nor the inclination to wade through the large treatises on mental disease which exist in our language; but there can be no excuse for their not studying this or some similar outline, and thereby keeping up their acquaintance with the subject. The book is admirably arranged and indexed. The second part contains sections on the various well-known types of insanity, as well as the more special forms of the disease. Treatment of the acute types for the most part must mean residence in an asylum, but Dr. Younger truly points out that home treatment is often successful in melancholia, and we may add that it is in this form, in its milder aspects at any rate, that complete recovery may be looked for. In acute mania recovery may follow; that is, recovery necessitating discharge from the asylum or from private medical treatment; but the psychological critic can nearly always detect some difference between the discharged patient and his fellow men. Not the least useful chapter in the book is that relating to the examination of lunatics for certification. Every practitioner upon whom this disagreeable duty may fall would do well to carefully study this chapter and make headings of it in his note-book before visiting his patient. Nine pages are devoted to the legal bearings of insanity, and useful hints are given. Elsewhere in the book the medical witness is cautioned not to attempt a definition of insanity; but we fear it is not easy to follow this advice in court when asked by judge or barrister what is meant by insanity. In these cases it is best to quote the definition of one or more well-known and admitted authorities, with, perhaps, the rider that, whatever else insanity may mean, it always means discord between the individual and his surroundings. We heartily commend this little work to our busy practitioners.

Golden Rules of Dental Surgery. By C. W. Glassington, M.R.C.S., L.D.S.Edin. (Bristol: John Wright and Co. Price 1s.).

Mr. Glassington makes no claim for any originality or research in his "Golden Rules of Dental Surgery," but his little booklet consists, as the title suggests, of a number of axioms encompassing the whole field of dental surgery. One feels that it would have been better had he specialised and confined his work to one aspect of dental surgery in so small a book. The section on Materia Medica and Therapeutics is perhaps the one which will be found most useful to students, and the author's advice as regards the treatment of patients is excellent, and practitioners as well as students would do well to bear it in mind.

